# Study protocol for a randomized controlled trial comparing penetrating needling and cognitive behavioral therapy for subjective tinnitus

**DOI:** 10.3389/fneur.2026.1877858

**Published:** 2026-09-18

**Authors:** Shasha Yang, Wanting Xu, Xuejun Guo, Qinwei Fu, Hao Liang, Qian Wang, Jing Wu

**Affiliations:** 1 Department of Otolaryngology, First Affiliated Hospital of Guizhou University of Traditional Chinese Medicine, Guiyang, Guizhou, China; 2 Guizhou University of Traditional Chinese Medicine, Guiyang, Guizhou, China; 3 Hospital of Chengdu University of Traditional Chinese Medicine, Chengdu, Sichuan, China; 4 Department of Health Research Methods, Evidence, and Impact (HEI), McMaster University, Hamilton, ON, Canada; 5 The Second Affiliated Hospital of Guizhou University of Traditional Chinese Medicine, Guiyang, Guizhou, China

**Keywords:** cognitive behavioral therapy, penetrating needling, protocol, randomized controlled trial, subjective tinnitus

## Abstract

**Objective:**

To describe the protocol for a randomized controlled trial designed to evaluate the clinical efficacy of penetrating needling compared with cognitive behavioral therapy (CBT) in patients with subjective tinnitus.

**Methods:**

This protocol describes a proposed assessor- and analyst-blinded, parallel-group randomized controlled trial. A total of 72 participants will be randomized 1:1 to a treatment group receiving penetrating needling plus health education or a control group receiving CBT plus health education. Penetrating needling will be administered five times weekly for two consecutive weeks (10 sessions). The primary outcomes are the Tinnitus Handicap Inventory (THI), the Tinnitus Questionnaire (TQ), and a Visual Analogue Scale (VAS) for tinnitus loudness. Secondary outcomes include the Pittsburgh Sleep Quality Index (PSQI), the Hamilton Depression Scale (HAMD), and the Hamilton Anxiety Scale (HAMA). Outcomes will be assessed at baseline, after one week of treatment, after two weeks of treatment, and at a 4-week follow-up (by telephone, online, or in clinic).

**Conclusion:**

As CBT is an established standard of care, this proposed parallel-group trial is designed to specifically evaluate the efficacy of penetrating needling. The findings of this protocol are expected to provide rigorous, high-quality evidence to determine the viability of penetrating needling as a neuromodulatory alternative for subjective tinnitus.

**Clinical trial registration:**

http://itmctr.ccebtcm.org.cn/, ITMCTR2024000264.

## 1 Introduction

Subjective tinnitus is defined as the perception of sound in the absence of external acoustic stimuli (1), and it affects approximately 15% of the general population (2). With population aging, its prevalence keeps increasing and imposes a substantial socioeconomic burden (3). Beyond auditory symptoms, patients often suffer from comorbid conditions such as depression, anxiety, sleep disturbances, and cognitive impairment (4, 5). The persistent nature of these phantom sounds typically triggers a vicious cycle of irritability and attentional deficits: emotional distress and symptom severity reinforce each other, significantly undermining patients’ overall quality of life (6, 7). Recent studies have continued to explore various therapeutic modalities for tinnitus, yet high-level evidence for non-pharmacological interventions (such as sound therapy, transcranial magnetic stimulation, and dietary supplements) remains limited (8).

While the exact pathophysiology of tinnitus has not yet been fully clarified, it is increasingly recognized as a disorder of maladaptive neuroplasticity. Alterations in functional connectivity between the insula and broader brain networks may disrupt the balance between auditory and non-auditory signaling, thereby sustaining phantom auditory perceptions (9). Mounting evidence indicates that tinnitus involves complex dysregulation in both auditory pathways and emotional networks (10), which highlights the need for therapeutic approaches that address both the auditory and limbic systems simultaneously.

However, current clinical management remains challenging. Pharmacotherapy and sound therapy are commonly used, but their efficacy varies across patient populations (11). Cognitive Behavioral Therapy (CBT) is currently recommended by multiple clinical guidelines as the gold standard for managing tinnitus-related distress (12). However, recent evidence has highlighted barriers to its widespread implementation, including limited accessibility and variable patient adherence. As a result, there is an urgent need for effective, more accessible alternative interventions.

Acupuncture has emerged as a promising intervention, with various clinical studies evaluating its effects (13, 14). Neuroimaging techniques—including functional magnetic resonance imaging (fMRI), electroencephalography (EEG), and positron emission tomography (PET)—have started to uncover the central mechanisms and neurochemical modulations induced by acupuncture in tinnitus patients (15). Strategic acupoint selection is critical to therapeutic outcomes (16), data mining shows that peri-auricular points are the most frequently used (17, 18).

Penetrating needling is a specialized advancement of traditional acupuncture techniques (19, 20). By integrating meridian theory with functional neuroanatomy, it adopts a three-dimensional needling strategy that targets specific acupoints on the ear and scalp, aiming to regulate local circulation and central neural pathways (21). Preliminary fMRI data suggest that this form of stimulation can modulate auditory-related brain regions, such as the basal ganglia, providing a neurophysiological basis for its use (22). For example, stimulating specific periauricular points like Tinggong (SI19) and Tinghui (GB2) is hypothesized to modulate functional connectivity between the auditory cortex and limbic networks. Despite these advancements, high-quality randomized controlled trial (RCT) protocols directly evaluating penetrating needling as an optimized technique remain lacking. Therefore, we designed this proposed RCT to rigorously assess the efficacy and safety of penetrating needling compared to the clinical standard (CBT) in patients with subjective tinnitus.

## 2 Methods and analysis

### 2.1 Protocol objectives

The first objective of this proposed study is to evaluate the effects of the 2-week penetrating needling on the Tinnitus Handicap Inventory (THI) (23, 24), the Tinnitus Questionnaire (TQ) (25), and the Visual Analogue Scale for tinnitus loudness (VAS) (26, 27). It is hypothesized that the penetrating needling program is more effective than CBT. The second objective of this proposed study is to evaluate the effects of the 2-week penetrating needling on the Pittsburgh Sleep Quality Index (PSQI) (28, 29), the Hamilton Depression Scale (HAMD) (30, 31), and the Hamilton Anxiety Scale (HAMA) (32, 33). It is hypothesized that the penetrating needling program is more effective than CBT in improving sleep quality, depression, and anxiety.

### 2.2 Protocol design

This protocol is a two-arm, parallel-group RCT with a 1:1 allocation ratio. A total of 72 eligible participants will be randomly assigned to the intervention group (Penetrating needling group) or the control group (CBT group), so that 36 participants will be included in each group. The study protocol has been approved by the Ethics Committee of The First Affiliated Hospital, Guizhou University of Traditional Chinese Medicine (approval No. KW-2024002), and any modifications will require a written application to the committee. The trial is registered with the International Traditional Medicine Clinical Trial Registry (ITMCTR) under registration number ITMCTR2024000264. The protocol will adhere to the rigorous standards outlined in the Declaration of Helsinki and the guidelines of the Consolidated Standards of Reporting Trials (CONSORT) (34), and the Standard Protocol Items: Recommendations for Interventional Trials (SPIRIT) (35). The feasibility of the proposed procedures, including patient tolerance of the penetrating needling technique and the recruitment workflow, has been confirmed through routine clinical practice at our institution prior to the initiation of this proposed trial.

We will recruit a total of 72 participants from the First Affiliated Hospital in Guiyang, China. The participants will be randomly allocated in a 1:1 ratio to either the penetrating needling group or the CBT group. The trial will consist of a 2-week intervention period followed by a 4-week follow-up period. Before randomization, we will assess the participants’ baseline characteristics. Outcome measures, including the THI, TQ, VAS, PSQI, HAMD, HAMA, and other indicators, will be assessed at the end of weeks 1, 2, and 4 (follow-up). The study procedure, including participant screening, randomization, intervention allocation, and follow-up assessments, is presented in the flow diagram (Figure 1). The schedule of enrolment, interventions, and assessments is shown in Figure 2.

**Figure 1:**
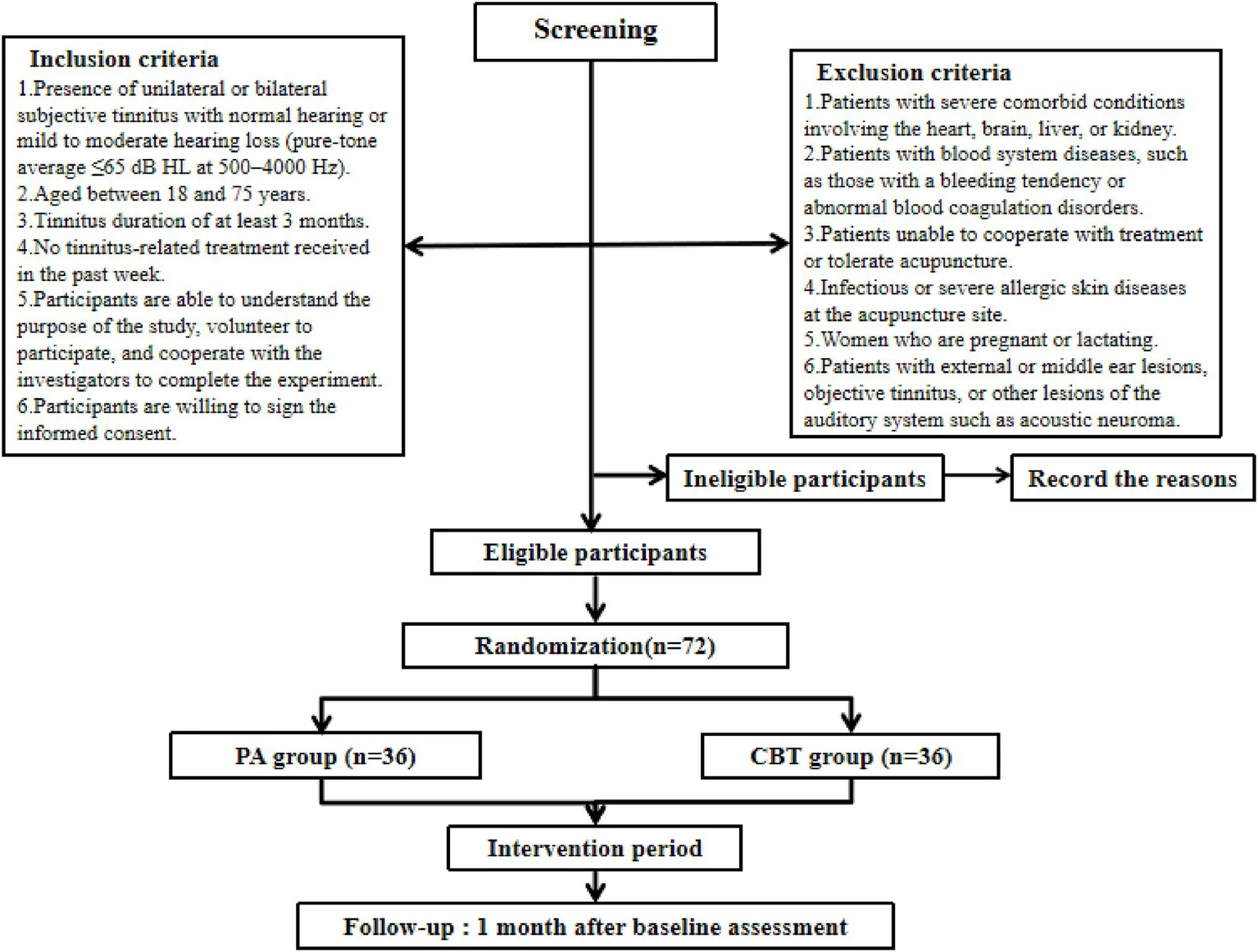
Flow diagram of the proposed protocol. This figure illustrates participant recruitment, randomization, intervention allocation, and follow-up assessments.

**Figure 2:**
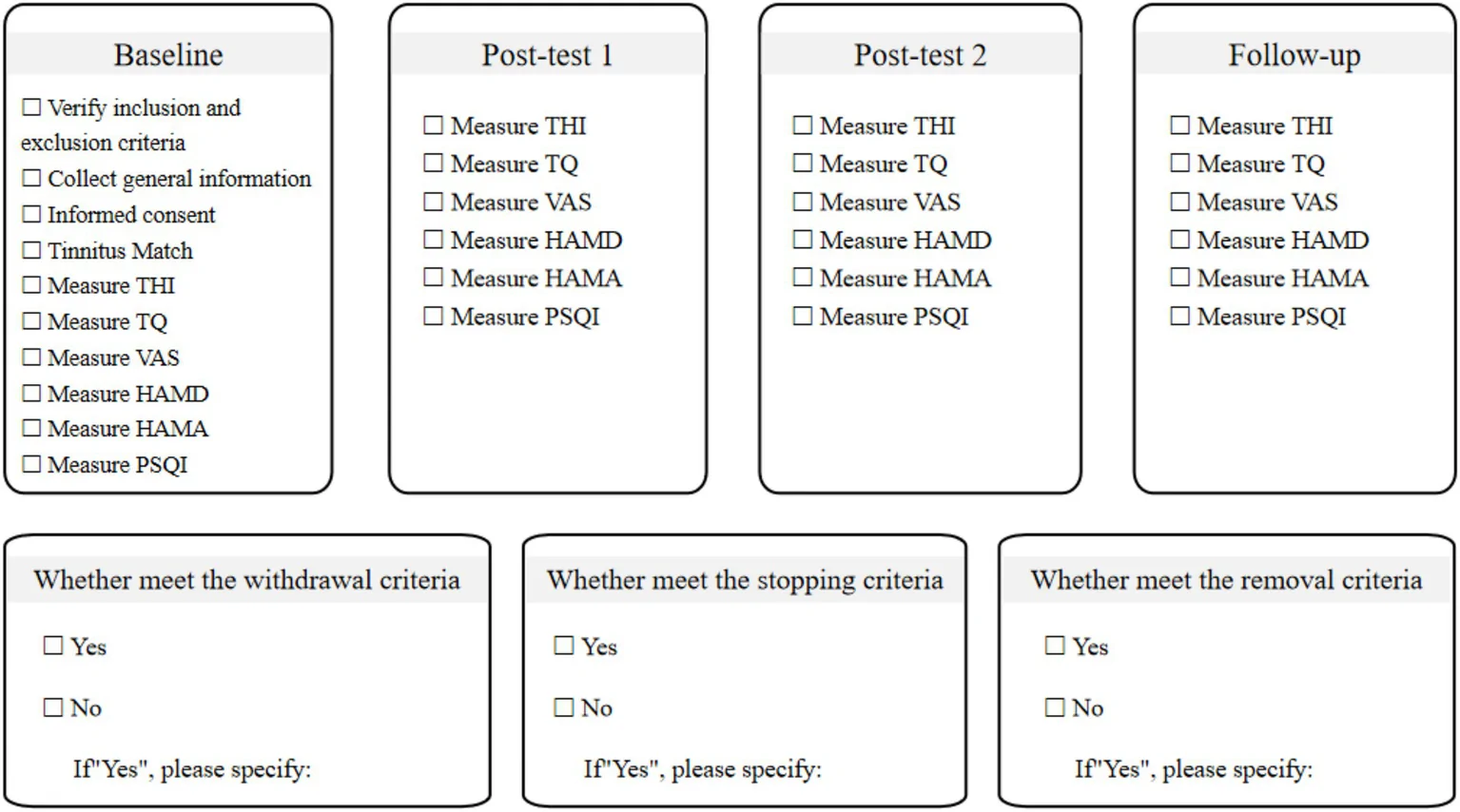
Schedule of enrolment, interventions, and assessments for the proposed trial.

### 2.3 Settings and participants

We will recruit participants for this trial through the Otolaryngology Outpatient Department of the First Affiliated Hospital of Guizhou University of Traditional Chinese Medicine. Recruitment advertisements will be posted in the department and disseminated via WeChat to reach potential participants. The Otolaryngology Department is a key discipline of the Guizhou Provincial Administration of Traditional Chinese Medicine. The estimated recruitment period will commence upon study approval and is expected to continue until the target sample size is achieved, with an anticipated completion date of December 2026. If case collection is insufficient by then, the period may be extended as needed. The trial registration will be updated to reflect any changes to the recruitment timeline. Interested individuals will contact the research team by phone or WeChat. The inclusion, exclusion, withdrawal, stopping, and removal criteria are described below.

### 2.4 Inclusion criteria

1. Presence of unilateral or bilateral subjective tinnitus with normal hearing or mild to moderate hearing loss (pure-tone average ≤65 dB HL at 500–4000 Hz).
2. Aged between 18 and 75 years.
3. Tinnitus duration of at least 3 months.
4. No tinnitus-related treatment (including pharmacotherapy, sound therapy, acupuncture, or CBT) received in the past 6 weeks (42 days) prior to enrollment.
5. Participants are able to understand the purpose of the study, volunteer to participate, and cooperate with the investigators to complete the experiment.
6. Participants are willing to sign the informed consent.

### 2.5 Exclusion criteria

1. Patients with severe comorbid conditions involving the heart, brain, liver, or kidney.
2. Patients with blood system diseases, such as those with a bleeding tendency or abnormal blood coagulation disorders.
3. Patients unable to cooperate with treatment or tolerate acupuncture.
4. Infectious or severe allergic skin diseases at the acupuncture site.
5. Women who are pregnant or lactating.
6. Patients with external or middle ear lesions, objective tinnitus, or other lesions of the auditory system such as acoustic neuroma.

### 2.6 Screening and verification

To ensure participants meet the inclusion and exclusion criteria, potential candidates will undergo a comprehensive baseline screening. Hearing thresholds and tinnitus characteristics will be assessed directly via pure-tone audiometry and tinnitus matching conducted by an audiologist. Comorbid conditions and exclusions will be verified through a detailed medical history interview and a review of the patients’ hospital electronic medical records. The study’s official WeChat account, used for recruitment dissemination, will be securely managed exclusively by the designated research coordinator.

### 2.7 Withdrawal criteria

Participants may withdraw from the study at any time without penalty.

### 2.8 Stopping criteria

Discontinuation for a specific participant will occurs if a serious adverse event (SAE) (defined as any untoward medical occurrence that results in death, is life-threatening, or requires inpatient hospitalization) occurs. Given the excellent safety profile of acupuncture, we do not expect any SAEs to occur.

### 2.9 Removal criteria

1. Intolerant to acupuncture treatment (defined as severe needle phobia, uncontrollable anxiety during the procedure, or unbearable pain leading to the patient’s refusal to continue).
2. Failure to complete the clinical trial for any reasons.
3. Request to withdraw due to loss of interest, scheduling conflicts, or personal reasons.
4. Adherence rate to the recommended treatment below 80% (fewer than 8 out of 10 sessions attended).

### 2.10 Allocation

An independent statistician will generate the random allocation sequence using SAS software (SAS Institute Inc., Cary, NC, USA) with block randomization (fixed block size of four, stratified by tinnitus laterality) to ensure equal numbers of participants to each group (36). Participants will be assigned to one of two treatment groups: “Penetrating needling” or “CBT.” The randomization list and block sizes will be concealed from the researchers. To ensure allocation concealment, sequentially numbered opaque sealed envelopes will be prepared by a staff member not involved in the study and stored in a locked cabinet. After obtaining informed consent and baseline data, the study physician will assign a unique study identification number to each participant and open the corresponding sealed envelope to reveal the assigned treatment group.

## 3 Description of penetrating needling intervention

The acupuncture intervention will be reported in accordance with the Standards for Reporting Interventions in Clinical Trials of Acupuncture (STRICTA) (37). All acupuncturists will receive standardized training on the protocol to ensure consistency and adherence. Acupoints are identified based on the National Standard of the People’s Republic of China (GB/T 12346–2021) (38).

The treatment protocol utilizes a total of 21 acupoints. To ensure reproducibility, the acupoints are divided into two fixed combinations that are administered alternately across sessions. The specific acupoints in each combination are detailed below and summarized in Table 1, with anatomical locations illustrated in Figures 3A–C.

**Table 1:** Proposed acupoint selection and needling parameters for the penetrating needling intervention.

Acupoint	Location	Needling depth (cun)^1^	Angle	Manipulation
Tinggong (SI19)	Anterior to the tragus, posterior to the condyloid process of the mandible	1–1.5	Perpendicular insertion	*Fameng* Needling Technique: the patient pinches their nose and inflates cheeks during needling to induce roaring sensation in the ear
Ermen (SJ21)	Anterior to the supratragic notch, posterior to the condyloid process of the mandible, slightly superior to Tinggong (SI19)	1.5–2	Perpendicular insertion	Slight opening of mouth
Tinghui (GB2)	Anterior to the intertragic notch, posterior to the condyloid process of the mandible, slightly inferior to Tinggong (SI19)	1–1.5	Perpendicular insertion	Slight opening of mouth
Erheliao (SJ22)	On the cephalic side, at the intersection of the sideburn and the anterior border of the auricle root	0.3–0.5	Penetrating Needling	Avoid arteries; Oblique insertion toward the center of the ear
Yifeng (SJ17)	Posterior to the ear lobe, in the depression anterior to the mastoid process	0.5–1	Penetrating Needling	Oblique insertion toward the center of the ear
Wangu (GB12)	On the head, in the depression posterior-inferior to the retroauricular mastoid	0.5–0.8	Penetrating Needling	Twirling reinforcing manipulation (<90° rotation)
Chimai (SJ18)	On the head, at the center of the mastoid process, at the junction of the middle and lower third of the line connecting Jiaosun (SJ20) and Yifeng (SJ17) along the curve of the helix	0.3–0.5	Penetrating Needling	Oblique insertion toward the center of the ear
Touqiaoyin (GB11)	On the head, posterior-superior to the mastoid process, at the junction of the upper two-thirds and lower third of the curved line connecting Tianchong (GB9) and Wangu (GB12)	0.5–0.8	Penetrating Needling	Oblique insertion toward the center of the ear
Luxi (SJ19)	On the head, at the junction of the upper and middle thirds of the curve between Jiaosun (SJ20) and Yifeng (SJ17) along the helix	0.3–0.5	Penetrating Needling	Oblique insertion toward the center of the ear
Yintang (DU29)	On the forehead, between the eyebrows	0.3–0.5	Perpendicular insertion	Lift and pinch local skin
Jiaosun (SJ20)	On the head. When the auricle is folded forward, the point is where the tip of the ear reaches the hairline	0.3–0.5	Penetrating Needling	Routine
Shuaigu (GB8)	On the head, 1.5 cun directly above the tip of the ear within the hairline, directly above Jiaosun (SJ20)	0.5–0.8	Penetrating Needling	Shuaigu (GB8) through Jiaosun (SJ20)
Tianchong (GB9)	On the head, 2 cun directly above the posterior border of the auricle root within the hairline and 0.5 cun behind Shuaigu (GB8)	0.5–0.8	Penetrating Needling	Tianchong (GB9) through Jiaosun (SJ20)
Baihui (DU20)	On the head, 5 cun directly above the anterior hairline, or at the intersection of the midline of the head and the line connecting the tips of the ears	0.5–0.8	Penetrating Needling	Baihui (DU20) through Sishencong (EX-HN1); Baihui (DU20) through Tongtian (BL7)
Sishencong (EX-HN1)	On the head, 1 cun anterior, posterior, and lateral to Baihui (DU20) (4 acupoints in total)	0.5–0.8	Penetrating Needling	Routine
Tongtian (BL7)	On the head, 4 cun directly above the anterior hairline on the midline, 1.5 cun lateral to the midline	0.3–0.5	Penetrating Needling	Routine
Shenting (DU24)	On the head, 0.5 cun directly above the anterior hairline on the midline	0.5–0.8	Penetrating Needling	Shenting (DU24) through Toulinqi (GB15)
Toulinqi (GB15)	On the head, 0.5 cun directly above the anterior hairline, at the pupil line, at the midpoint of the line between the Shenting (DU24) and the Touwei (ST8)	0.5–0.8	Penetrating Needling	Routine

^1^Cun is a traditional acupuncture unit of measurement; 1 cun ≈ width of the patient’s thumb at the interphalangeal joint.

**Figure 3:**
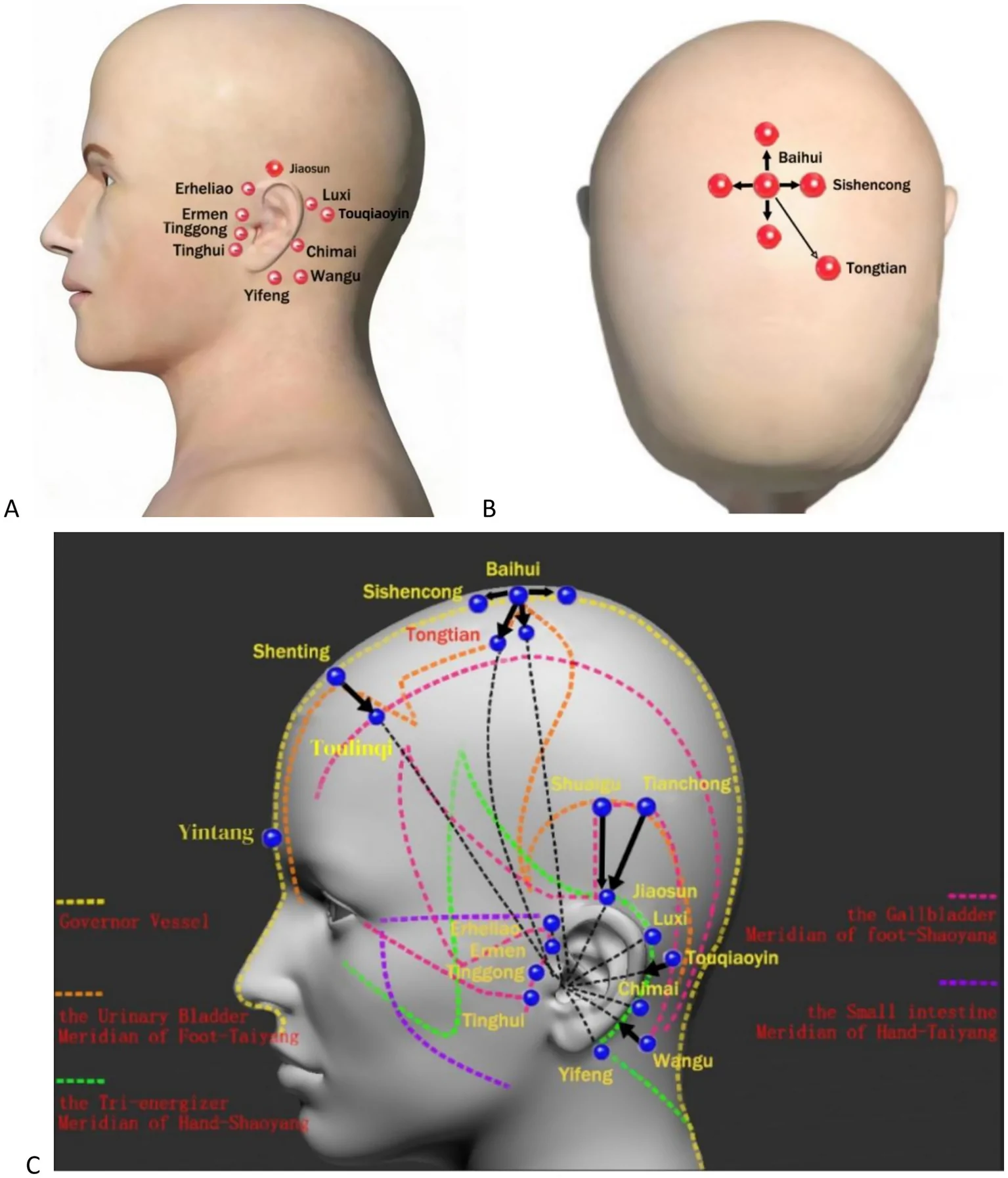
Location of periauricular acupoints **(A–C)**.

Combination A includes Tinggong (SI19) with the *Fameng* technique, plus five peri-auricular points (Ermen (SJ21), Tinghui (GB2), Yifeng (SJ17), Wangu (GB12), Erheliao (SJ22)) and five cranial points (Baihui (DU20) toward Sishencong (EX-HN1), Shenting (DU24) toward Toulinqi (GB15), Yintang (DU29), Shuaigu (GB8) toward Jiaosun (SJ20), Tianchong (GB9) toward Jiaosun (SJ20)).

Combination B includes the same Tinggong (SI19) with the Fameng technique, plus five peri-auricular points (Chimai (SJ18), Touqiaoyin (GB11), Luxi (SJ19), and the remaining points from the ear region) and five cranial points (Baihui (DU20) toward Tongtian (BL7), plus the other four cranial points not used in Combination A). The two combinations are applied on odd-numbered and even-numbered sessions, respectively. Each session uses ten points in total. A diagram showing the acupoints is provided in Figures 3A–C. The treatment will be administered five times weekly for two consecutive weeks, totaling ten sessions. Each session lasts 20 min. Disposable sterile needles (*Huo Tuo* brand; 0.20 mm × 25 mm for facial points, 0.30 mm × 40 mm for auricular points) will be used. Following insertion, all points will be manipulated to elicit *Deqi* (39)—a characteristic sensation of numbness, tingling, or heaviness indicative of effective activation. For Tinggong (SI19), the Fameng technique requires the patient to perform a maneuver similar to the Valsalva maneuver (pinching the nose, holding the breath, and inflating the cheeks) during needle insertion. Successful application is confirmed by the patient’s perception of a sizzling or roaring sound in the affected ear. All needles will be retained for 20 min, and the entire 2-week intervention period will be followed by a one-month follow-up assessment.

### 3.1 Cognitive behavioural therapy control group

Both the penetrating needling group and the control group will receive standardized health education. This consists of a 15-min introductory session and an informational booklet covering basic auditory anatomy, mechanisms of tinnitus, lifestyle recommendations, and general dietary advice.

The control group will additionally receive a standardized, clinician-led CBT program as an active comparator. To maintain methodological rigor and minimize attention bias, this intervention will be delivered in-person within a dedicated psychological counseling room within the hospital’s outpatient department. The intervention will be delivered by a clinical psychologist or an attending otolaryngologist who has received certified training in CBT specifically for tinnitus.

Unlike the basic health education which passively provides lifestyle advice, the CBT program actively utilizes psychological techniques. The program begins with a foundational psychoeducation component, where participants learn about the biological, psychological, and social determinants of tinnitus through individual face-to-face interviews and visual aids (pictures and videos). This stage is designed to address maladaptive beliefs and foster a proactive approach to managing tinnitus-related distress. The core intervention comprises sleep hygiene education, relaxation training, imagery techniques, and cognitive restructuring, all tailored to the participant’s specific needs. Throughout the study, participants will be required to maintain a daily thought record to document tinnitus-related emotions and negative automatic thoughts. During each scheduled 20-min session, the investigator will review these records with the patient to identify and modify negative cognitions through structured clinical dialog.

To ensure equivalent clinical attention and therapeutic engagement, the CBT intervention will be administered five times weekly for two consecutive weeks (ten sessions in total). This rigorous design ensures that any observed differences in outcomes between the two groups can be attributed to the specific effects of the interventions rather than disparities in the frequency of clinical contact or the treatment environment.

### 3.2 Intervention integrity

To ensure protocol fidelity, a standardized checklist will be used to track participant progress from baseline through the week 4 follow-up. We will document screening, enrollment, and completion rates in accordance with CONSORT guidelines.

Attendance for both the penetrating needling and CBT groups will be recorded in a dedicated log. To ensure data quality, an adherence rate of at least 80% (8 out of 10 sessions) is required for inclusion in the per-protocol analysis. Additionally, participants will receive reminders via telephone or WeChat for all scheduled sessions and follow-up assessments to minimize attrition and ensure high-quality data collection.

### 3.3 Sample size calculations

The sample size was calculated based on the primary outcome, the THI score. The standard deviation (*σ*) of the THI score change was estimated to be 12.0, and the minimal clinically important difference (MCID) between the two groups was set at 8.0 points, based on previous literature (40). Using a two-sided significance level of 5% (*α* = 0.05, Zα/2 = 1.96) and a power of 80% (*β* = 0.20, Zβ = 0.84), the sample size per group was calculated using the formula for comparing two independent means. The formula is shown below (41).

$n=\frac{2{\left(Z_{\alpha /2}+Z_{\beta }\right)}^{2}\sigma^{2}}{\delta^{2}}$

Based on the formula, the calculated sample size is 29 cases per group. Accounting for a 20% dropout rate, we determined that 36 participants per group are required, resulting in a total sample size of 72 participants.

### 3.4 Assessments and time points

Assessments will be performed at baseline and at each protocol-defined time point throughout the study until completion. Participants will be assessed at four time points: baseline, week 1, week 2, and the 4-week follow-up. The timing of assessments is shown in Figure 2, and the corresponding outcome measures are detailed in Table 2.

**Table 2:** Spirit schedule of enrolments, intervention and assessments of the protocol.

Time points	Baseline	Week 1	Week 2	Week 4
Enrollment				
Verify inclusion and exclusion criteria	×			
Informed consent	×			
Tinnitus Match	×			
Allocation	×			
Interventions				
Penetrating needling group	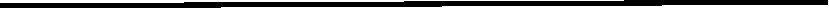			
CBT group	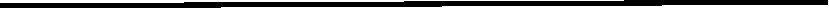			
Assessments				
THI	×	×	×	×
TQ	×	×	×	×
VAS	×	×	×	×
PSQI	×	×	×	×
HAMD	×	×	×	×
HAMA	×	×	×	×
Adherence assessment		Keep track		
Safety assessment		keep track		

CBT, Cognitive Behavioral Therapy; THI, Tinnitus Handicap Inventory; TQ, Tinnitus Questionnaire; VAS, Visual Analogue Scale for tinnitus loudness; PSQI, Pittsburgh Sleep Quality Index; HAMD, Hamilton Depression Scale; HAMA, Hamilton Anxiety Scale; SPIRIT, Standard Protocol Items, Recommendations for Interventional Trials.

### 3.5 Blinding

Due to the inherent nature of the interventions (penetrating needling vs. CBT), blinding of participants and practitioners is not feasible. To minimize detection and attrition bias, this trial adopts an assessor-blinded and analyst-blinded design.

Outcome assessments, including the collection of post-test and follow-up data, will be conducted by independent assessors who are not involved in treatment delivery and are unaware of the group assignments. Similarly, the statistical analyst will remain blinded to the allocation codes throughout the data processing phase. Group assignments will only be revealed after the primary analysis is finalized to ensure the objectivity and scientific integrity of the results.

## 4 Outcomes

### 4.1 Primary outcomes

The primary outcomes include the Tinnitus Handicap Inventory (THI), Tinnitus Questionnaire (TQ), and Visual Analogue Scale (VAS). Data will be collected at four time points: baseline, after 1 and 2 weeks of treatment, and at the 4-week follow-up.

1. Tinnitus Handicap Inventory (THI): A standardized 25-item self-report questionnaire designed to assess the multi-dimensional impact of tinnitus on quality of life. It evaluates functional, emotional, and catastrophic responses. The total score (0–100) reflects the degree of handicap: slight (0–16), mild (18–36), moderate (38–56), severe (58–76), and catastrophic (78–100).
2. Tinnitus Questionnaire (TQ): A validated self-assessment tool comprising 52 items scored on a 3-point scale (0 = true, 1 = partly true, 2 = not true). The total score ranges from 0 to 84, providing a composite score that quantifies tinnitus-related psychological distress, with higher scores indicating greater distress across multiple subscales, including emotional distress, cognitive distress, and auditory perceptual difficulties.
3. Visual Analogue Scale (VAS): A subjective 10-point linear scale used to quantify tinnitus loudness. Participants rate their perceived intensity from 0 (“no tinnitus”) to 10 (“extremely loud tinnitus”). This scale provides a sensitive measure for tracking changes in symptom intensity over time.

### 4.2 Secondary outcomes

Secondary outcomes focus on emotional well-being and sleep quality, measured at baseline, after 1 and 2 weeks of treatment, and at the 4-week follow-up.

1. Pittsburgh Sleep Quality Index (PSQI): A 19-item self-rated instrument assessing sleep quality and disturbances. It generates seven component scores (sleep latency, duration, and efficiency), which are summed to yield a global score (0–21).
2. Hamilton Depression Scale (HAMD-24): A clinician-administered scale for quantifying the severity of depressive symptoms. It covers 24 items, including mood, suicidal ideation, and somatic symptoms. Higher scores indicate more pronounced depressive states.
3. Hamilton Anxiety Scale (HAMA): A clinician-administered assessment designed to quantify anxiety severity across 14 categories, with each item rated on a 0–4 scale. The total score reflects the overall level of psychic and somatic anxiety.

### 4.3 Safety assessment

Safety will be assessed by recording all adverse events (AEs) occurring during the trial. AEs will be graded according to the CTCAE version 5.0. Specific attention will be paid to acupuncture-related risks, including local hematoma, needle pain, and syncope (fainting). All events will be documented on standardized record forms, and incidence rates will be calculated for both groups. Emergency unblinding is permitted only for serious adverse events (SAEs) where knowledge of the treatment assignment is clinically necessary for participant management.

### 4.4 Data collection, management, and monitoring

The recruitment period will commence following protocol approval and is expected to continue until the target sample size of 72 participants is achieved, with an anticipated completion date of December 2026. If the target sample size is not achieved by this date, the period may be extended. Quantitative data, including demographic characteristics, clinical assessments (pure-tone audiometry and tinnitus evaluation), and validated outcome scales, will be systematically documented in Case Report Forms (CRFs).

The EpiData management system will be utilized for electronic data storage. To ensure accuracy, independent double data entry and cross-verification will be performed by two researchers. A random audit of 10% of the CRFs against the database will be conducted periodically to identify and rectify discrepancies. Data quality and study progress will be monitored by the Ethics Committee of The First Affiliated Hospital, Guizhou University of Traditional Chinese Medicine, ensuring adherence to the approved protocol and GCP guidelines.

### 4.5 Data analysis

#### 4.5.1 Statistical analysis

Statistical analysis will be performed using SAS 9.4. The primary analysis will follow the intention-to-treat (ITT) principle, including all randomized participants. A per-protocol (PP) analysis will also be conducted for participants who strictly adhered to the protocol, including high compliance (≥60% attendance), no use of prohibited medications, and complete CRF data.

Continuous variables (THI, TQ, and VAS scores) will be presented as means ± standard deviations or medians (interquartile ranges). Comparisons between the two groups at each time point will be conducted using independent t-tests or Mann–Whitney U tests, depending on data normality. To account for repeated measurements (baseline, weeks 1, 2, and 4), mixed-effects models will be employed as the primary analytic approach to assess treatment effects over time. These models are well-suited for this design as they handle missing data under the missing-at-random assumption, accommodate individual variability through random intercepts and slopes, and are robust to irregularly spaced time points. Fixed effects will include time, group, and the time-by-group interaction term to evaluate differences in the trajectory of change between the two intervention groups. Generalized Estimating Equations (GEE) will be used as a sensitivity analysis to assess the robustness of findings under a population-averaged approach. The choice of the primary analytic model was pre-specified to maintain methodological integrity.

Categorical data will be analyzed using the chi-squared test or Fisher’s exact test. For ordinal data, the Kruskal-Wallis test will be used for overall comparisons, followed by Wilcoxon rank-sum tests for pairwise comparisons. Multiple imputation will be utilized to handle missing data. Sensitivity analyses will be conducted to assess the robustness of findings under different missing data assumptions. All tests will be two-sided, with a significance level of *α* ≤ 0.05.

#### 4.5.2 Qualitative assurance

To ensure protocol integrity, all researchers will undergo standardized training on participant recruitment, randomization, intervention protocols, and CRF completion. Acupuncturists must possess a valid license and at least 5 years of clinical experience to minimize performance bias. The Ethics Committee of The First Affiliated Hospital, Guizhou University of Traditional Chinese Medicine, will provide independent oversight of study implementation and participant safety. All committee members declare no conflicts of interest regarding this trial.

## 5 Discussion

This protocol describes a proposed RCT to directly compare penetrating needling with CBT for subjective tinnitus. CBT is currently the gold standard for managing tinnitus-related distress. However, its widespread implementation can be constrained by the need for specialized clinical resources and significant time commitments. Pharmacological and sound therapies often offer only limited relief. While acupuncture shows promise, high-quality evidence specifically for penetrating needling remains scarce. Our proposed trial aims to fill this gap by evaluating a two-week intensive penetrating needling regimen aimed at modulating auditory and limbic pathways. To ensure a fair comparison, the CBT group receives an equal number of sessions of the same duration.

Key strengths of this study include blinding of both assessors and analysts, the use of validated outcome measures (THI, TQ, VAS, PSQI, HAMD, HAMA), and a sample size calculated to detect clinically meaningful changes in THI scores. However, several limitations should be noted: the study is single-center with a modest sample size (n = 72), and blinding of participants and acupuncturists is unavoidable, which may introduce performance bias. Additionally, the four-week follow-up period—chosen to balance practical constraints (including funding limitations and participant retention) with the need for rigorous data collection—means longer-term effects remain unclear, which limits the generalisability of our findings. Furthermore, while the THI and TQ are well-validated and widely used instruments that facilitate cross-study comparisons, they place relatively strong emphasis on emotional distress and catastrophizing—domains that are directly targeted by CBT. This could potentially favor the CBT group. Additionally, while the one-week washout period was initially chosen to facilitate recruitment, future studies would benefit from a longer washout period (6 weeks or more) to minimize confounding from prior treatments. Future trials should include extended follow-up periods (6 or 12 months) and systematically assess whether participants have sought additional treatments during the post-intervention period.

Recent developments in neuromodulation and personalized medicine have renewed interest in non-pharmacological tinnitus interventions, underscoring the relevance of this comparative trial. If penetrating needling proves more effective than CBT, it will serve as a practical alternative for patients who cannot access or do not benefit from psychological therapy. Future trials should include multiple centers and longer follow-up periods to strengthen the evidence base. Overall, this rigorous proposed RCT protocol is designed to provide valuable evidence on the efficacy and safety of penetrating needling for subjective tinnitus. Results are expected to be available in 2027.

## 6 Ethics and dissemination

This clinical trial will be conducted in strict accordance with the Declaration of Helsinki and the International Ethical Guidelines for Health-related Research Involving Humans. The protocol, informed consent forms, and recruitment materials have been approved by the Ethics Committee of The First Affiliated Hospital, Guizhou University of Traditional Chinese Medicine (Approval No. KW-2024002). Any subsequent modifications to the protocol will be submitted to the committee for formal review and approval.

Prior to enrollment, the study’s objectives, procedures, and potential risks will be explained in detail to each participant by a designated investigator. Written informed consent will be obtained from all participants in a private clinical setting before any baseline assessments. Upon completion of the study, findings will be disseminated through publication in peer-reviewed journals, presentations at international conferences, and reporting via clinical trial registries. De-identified individual participant data will be made available upon reasonable request from the corresponding author following the primary publication.
